# The Anthocyanin Delphinidin 3-Rutinoside Stimulates Glucagon-Like Peptide-1 Secretion in Murine GLUTag Cell Line via the Ca^2+^/Calmodulin-Dependent Kinase II Pathway

**DOI:** 10.1371/journal.pone.0126157

**Published:** 2015-05-11

**Authors:** Masaki Kato, Tsubasa Tani, Norihiko Terahara, Takanori Tsuda

**Affiliations:** 1 College of Bioscience and Biotechnology, Chubu University, Kasugai, Aichi, Japan; 2 Department of Food Science and Technology, Minami-Kyushu University, Miyazaki, Japan; University of Toronto, CANADA

## Abstract

Glucagon-like peptide-1 (GLP-1) is an incretin hormone secreted from enteroendocrine L-cells. Although several nutrients induce GLP-1 secretion, there is little evidence to suggest that non-nutritive compounds directly increase GLP-1 secretion. Here, we hypothesized that anthocyanins induce GLP-1 secretion and thereby significantly contribute to the prevention and treatment of diabetes. Delphinidin 3-rutinoside (D3R) was shown to increase GLP-1 secretion in GLUTag L cells. The results suggested that three hydroxyl or two methoxyl moieties on the aromatic ring are essential for the stimulation of GLP-1 secretion. Notably, the rutinose moiety was shown to be a potent enhancer of GLP-1 secretion, but only in conjunction with three hydroxyl moieties on the aromatic ring (D3R). Receptor antagonist studies revealed that D3R-stimulates GLP-1 secretion involving inositol 1,4,5-trisphosphate receptor-mediated intracellular Ca^2+^ mobilization. Treatment of GLUTag cells with a Ca^2+^/calmodulin-dependent kinaseII (CaMKII) inhibitor (KN-93) abolished D3R-stimulated GLP-1 secretion. In addition, treatment of GLUTag cells with D3R resulted in activation of CaMKII. Pre-treatment of cells with a G protein-coupled receptor (GPR) 40/120 antagonist (GW1100) also significantly decreased D3R-stimulated GLP-1 secretion. These observations suggest that D3R stimulates GLP-1 secretion in GLUTag cells, and that stimulation of GLP-1 secretion by D3R is mediated via Ca^2+^-CaMKII pathway, which may possibly be mediated by GPR40/120. These findings provide a possible molecular mechanism of GLP-1 secretion in intestinal L-cells mediated by foods or drugs and demonstrate a novel biological function of anthocyanins in regards to GLP-1 secretion.

## Introduction

Glucagon-like peptide-1 (GLP-1) secreted from enteroendocrine L-cells is one type of incretin and stimulates glucose-dependent insulin secretion and proliferation of pancreatic β-cells [1–3]. Due to its established role in the metabolic response, particularly glucose homeostasis, GLP-1 is an important factor in the treatment and prevention of type 2 diabetes. Several therapeutic approaches to enhance GLP-1 action are being studied and include the use of GLP-1 analogs, which improve glycemic control in type 2 diabetes patients [4, 5]. However, GLP-1 analogs are not suitable for oral administration and must be hypodermically injected. As circulating GLP-1 is rapidly inactivated by the enzyme dipeptidyl peptidase IV (DPP-4) through cleavage of the N-terminal region of intact GLP-1 [6, 7], DPP-4 inhibitors are promising therapeutic agents for extending the half-life of endogenously secreted GLP-1. To date, several DPP-4 antagonists have been identified that ameliorate hyperglycemia in type 2 diabetes patients [8, 9].

These approaches are effective for controlling blood glucose levels in type 2 diabetic patients. However, an alternative therapeutic approach is to increase endogenous GLP-1 secretion through modulation of the secretory mechanisms in intestinal L cells using pharmaceutical agents or dietary factors. This novel therapeutic strategy may help treat diabetes and decrease the required doses of other diabetic medicines. A number of nutrients and small molecules are reported to increase GLP-1 secretion *in vitro* and *in vivo* and include certain fatty acids [10–12], as well as glutamine and arginine, which are well-characterized GLP-1 secretagogues [13–15]. Protein hydrolysates have also been reported to induce enhanced GLP-1 secretion [16–19].

We previously demonstrated that curcumin, a yellow pigment isolated from turmeric, markedly increases GLP-1 secretion in the murine GLUTag cell line [20]. Despite the abundant evidence that several nutrients and drug candidates stimulate GLP-1 secretion, there is little evidence that non-nutritive food compounds, and not the nutrients themselves, are able to directly enhance GLP-1 secretion.

**Fig 1:**
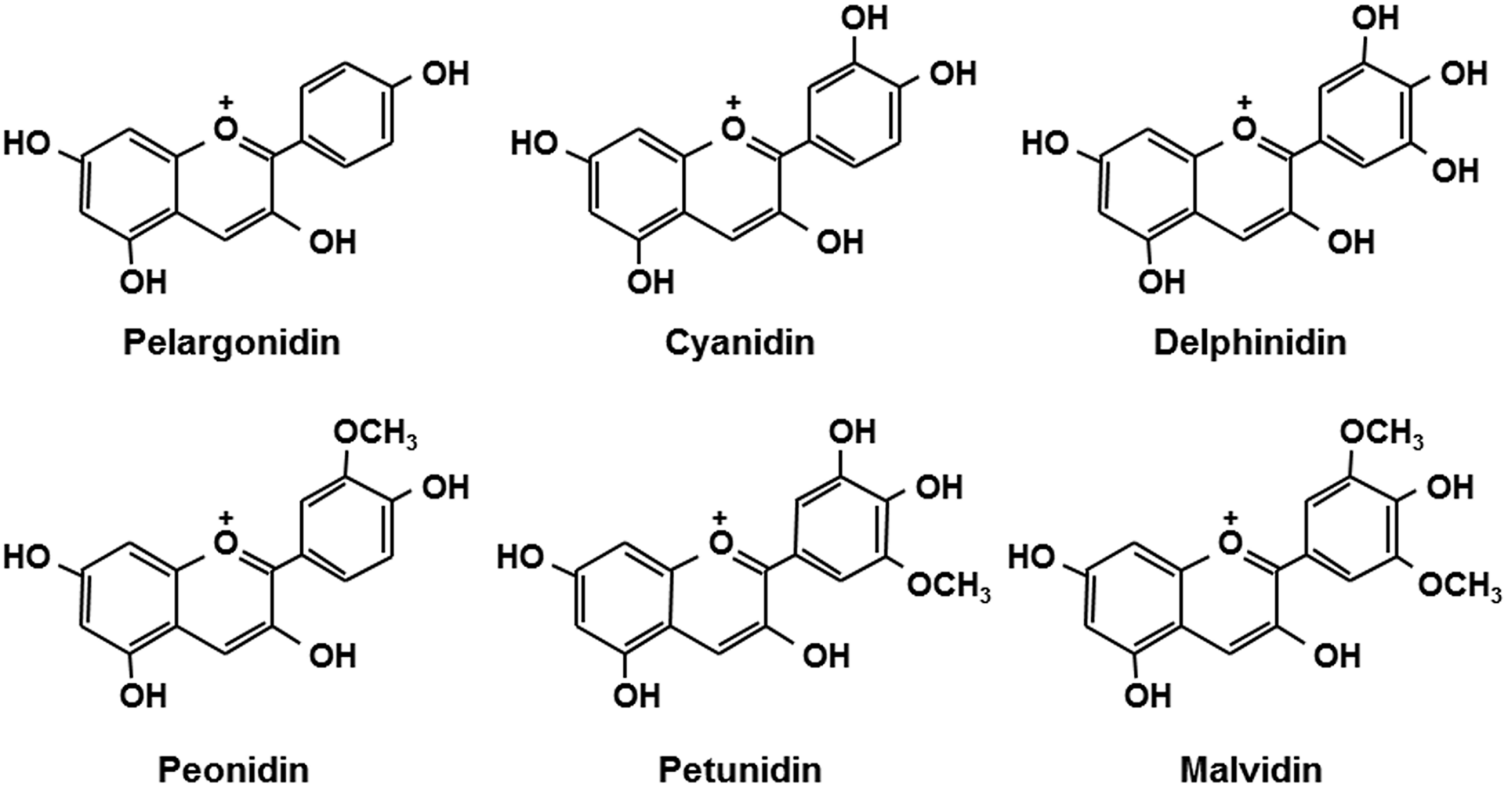
Chemical structure of anthocyanidins.

Anthocyanins are flavonoid phytopigments [21] that are found naturally in plants in the form of glycosides and are widely available in fruits and vegetables commonly consumed by humans. Recent research suggests that the consumption of anthocyanin-rich foods is associated with various health benefits [21–24]. Our research group demonstrated that anthocyanin-rich extract (bilberry and black soybean) reduces blood glucose levels and improves insulin sensitivity in type 2 diabetic mice [25, 26]. A recent epidemiological study showed that a higher consumption of anthocyanins and anthocyanin-rich fruit is associated with a lower risk of type 2 diabetes [24]. The molecular mechanism underlying this effect can be explained by the activation of AMP-activated protein kinase [25, 26]. However, at least six principal types of anthocyanidins have been identified to date (Fig 1), and many anthocyanins can be derived from these six types through structural modifications, such as the addition of substituent groups on the B ring, conjugation of various types and numbers of sugars, and the presence or absence of an acyl group. For this reason, anthocyanins may have various anti-diabetic effects via mechanisms other than AMP-activated protein kinase activation, such as the modification of GLP-1 activity. We hypothesized that anthocyanins facilitate GLP-1 secretion and thereby contribute to the prevention and treatment of diabetes.

In the present study, we demonstrated that delphinidin 3-rutinoside (D3R) significantly increased GLP-1 secretion in GLUTag cells, and clarified the structure-activity relationship using anthocyanin derivatives. Moreover, this increase was found to involve the inositol 1,4,5-trisphosphate receptor (IP_3_R)-mediated intracellular Ca^2+^ mobilization-Ca^2+^/calmodulin-dependent kinase II (CaMKII) pathway.

## Materials and Methods

### Chemicals

The purity of all administered chemicals was over 98%. Commercially available purified anthocyanins (pelargonidin, Pel; pelargonidin 3-glucoside, P3G; cyanidin, Cy; cyanidin 3-glucoside, C3G; cyanidin 3-rutinoside, C3R; delphinidin, Del; delphinidin 3-glucoside, D3G; D3R; peonidin, Peo; peonidin 3-glucoside, Peo3G; peonidin 3-rutinoside, Peo3R; Petunidin, Pet; petunidin 3-glucoside, Pet3G; malvidin, Mal; malvidin 3-glucoside, Mal3G; and malvidin 3,5-diglucoside, Mal3,5dG) were obtained from Tokiwa Phytochemical (Chiba, Japan) and Extrasynthèse (Genay, France). Malvidin 3-rutinoside (Mal3R, > 96% purity) was isolated and purified from tuber dry powder of black ginger (*Kaempferia parviflora* L.) using preparative ODS-HPLC. The structure of isolated Mal3R was verified by MS and NMR. The chemical structures of these anthocyanins are shown in Fig 1. Forskolin (Fos), ionomycin, 3-isobutyl-1-methylxanthine (IBMX), sodium dantrolene, verapamil chloride, H-89, KN-93, and Gö6983 were obtained from Wako Pure Chemical Industries (Osaka, Japan). 2-Aminoethyl diphenylborinate (2-APB) and GW1100 were purchased from Cayman Chemical (Ann Arbor, MI). NF449 was obtained from Abcam (Cambridge, MA). Rabbit polyclonal phospho-CaMKII (Thr286) antibody (#3361), rabbit monoclonal CaMKII antibody (#4436), and rabbit polyclonal β-actin antibody (#4967) were purchased from Cell Signaling Technology (Beverly, MA). BAPTA-AM and PD98059 were obtained from Merck (Darmstadt, Germany).

### Cell culture and GLP-1 secretion

The murine GLUTag L cell line [27] (a gift from Dr. D. J. Drucker, University of Toronto, Toronto, Canada) was routinely cultured in Dulbecco’s modified Eagle’s medium containing 25 mM glucose supplemented with 10% fetal bovine serum at 37°C in a humidified atmosphere containing 5% CO_2_. For secretion experiments, once the cells reached 80% confluence after seeded on 24-well plates at a density of 2 x 10^5^ cells/well, the medium was replaced with glucose free Krebs-Ringer bicarbonate buffer (120 mM NaCl, 5 mM KCl, 2 mM CaCl_2_, 1 mM MgCl_2_, and 22 mM NaHCO_3_) supplemented with 0.5% (w/v) fatty acid-free BSA to starve the cells for 1 h. The cells were then incubated with various test compounds in glucose free Krebs-Ringer bicarbonate buffer containing 0.5% fatty acid-free BSA for 2 h [20]. After treatment, the medium was collected and centrifuged at 800 × g for 5 min at 4°C to remove any floating cells. Secreted GLP-1 was assayed using an ELISA specific for GLP-1(7–36 amide) and GLP-1(7–37) (GLP-1 ELISA-kit, Millipore, St. Charles, MS) according to the manufacturer’s instructions.

### GLUTag cell treatment and western blotting

The medium of cultured GLUTag cells was replaced with serum-free Dulbecco’s modified Eagle’s medium containing 1% BSA for 3 h. After incubation, the cells were treated with vehicle (0.1% DMSO) or D3R for the indicated time periods and conditions and then lysed [28]. Supernatant aliquots were treated with Laemmli sample buffer for 5 min at 100°C [29], and the samples (20 μg protein) were separated by SDS-PAGE. After electrophoresis, proteins were transblotted onto nitrocellulose membranes and probed with various primary antibodies for 16 h at 4°C. The proteins were then reacted with horseradish peroxidase-conjugated anti-rabbit antibody, and immunoreactivity was visualized using Pierce Western Blotting Substrate (Thermo Fisher Scientific, Yokohama, Japan). The relative density of the stained proteins was evaluated using Multi Gauge Ver 3.0 Densitograph Software (Fuji Film, Tokyo, Japan).

### Measurement of intracellular cAMP levels

Cells were extracted by treatment with 0.1 M HCl to avoid degradation of cAMP, and the cAMP levels of the extract were measured using a cyclic AMP EIA kit (Cayman Chemical, Ann Arbor, MI) according to the manufacturer’s instructions.

### Statistical analysis

All data are expressed as the means ± SEM. Differences in the GLP-1 secretion or cytosolic cAMP concentrations of GLUTag cells were compared by the Tukey-Kramer test. In other cases, the data were analyzed by two-way ANOVA. If the interaction effect of two components (D3R and inhibitor) was significant, the Tukey-Kramer test was performed to compare the differences between the groups. For all statistical tests, values without a common letter (a, b, c, d and e) on the bar graphs are significantly different at *P* values < 0.05.

## Results

### D3R significantly stimulates GLP-1 secretion from GLUTag cells

**Fig 2:**
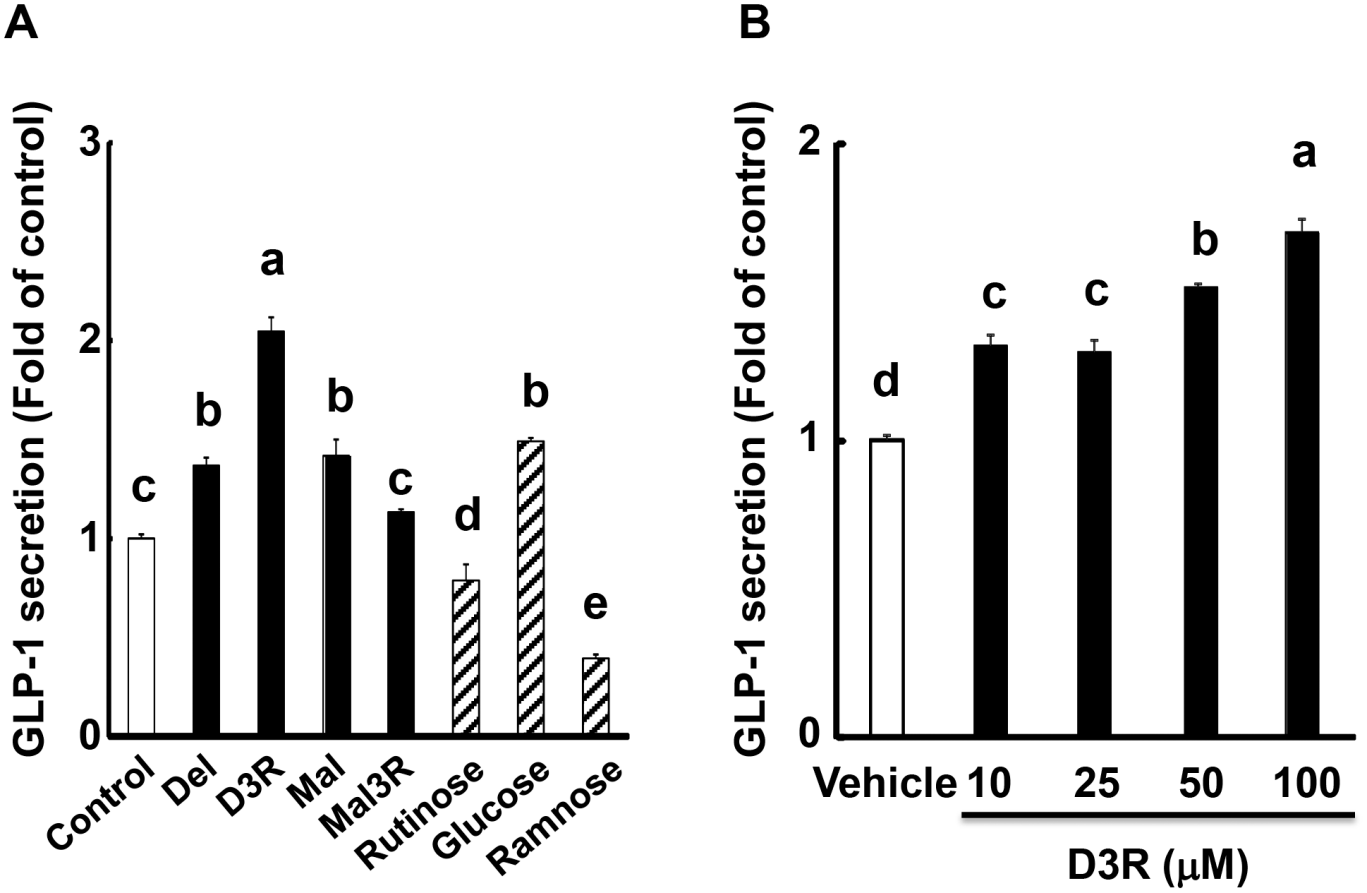
GLP-1 secretion in the medium of GLUTag cells treated with various anthocyanins. All examined (A) anthocyanins (100 μM) and sugars (rutinose, glucose, and rhamnose; 100 μM), or (B) varying concentrations of D3R were administered for 2 h. The GLP-1 concentration in the medium was then determined by ELISA. Secreted GLP-1 levels are expressed as the fold change of the control levels (= 1.0). Values are expressed as the means ± SEM, n = 3–9. Values without a common letter (a, b, c, d, and e) are significantly different at *P* < 0.05 (Tukey-Kramer test).

We first screened various types of anthocyanins for their ability to stimulate GLP-1 secretion in GLUTag cells. The screening revealed that three anthocyanins, D3R, which contains three B-ring hydroxyl moieties and rutinose, Del (the aglycone of D3R), and Mal, which has two methoxyl moieties on the B-ring, resulted in a significant increase in the secretion of GLP-1 by GLUTag cells (Fig 2A). Notably, however, the GLP-1 secretion induced by D3R was markedly higher compared with that induced by Del and Mal treatment. In addition, D3R stimulated GLP-1 secretion in a concentration-dependent manner (Fig 2B). Treatment of GLUTag cells with 10 μM D3R was sufficient to significantly stimulate GLP-1 secretion. However, we administered a final concentration of 100 μM of D3R to obtain clearer measurements of the inhibitory effects in the cell signaling pathway experiments (Fig 2B). Interestingly, glucosyl anthocyanins (P3G, C3G, D3G, Peo3G, Pet3G, Mal3G, and Mal3,5dG) did not significantly increase GLP-1 secretion (data not shown). Additionally, the other examined aglycones (Pel, Cy, Peo, and Pet) and rutinosyl anthocyanins (C3R, Peo3R, and Mal3R) had no detectable effect on GLP-1 secretion. In addition, we confirmed that rutinose itself did not stimulate GLP-1 secretion (Fig 2A), and that the treatment of GLUTag cells with up to 100 μM of each tested anthocyanin for 2 h had no cytotoxic effects, as shown by a cell viability >98% based on analysis using an automatic live cell counter system (Countess, Life Technologies, Tokyo, Japan) (data not shown). Based on the results of this initial screening, D3R was selected for further studies of the GLP-1 secretion mechanism.

### D3R-induced GLP-1 secretion is regulated by endogenous Ca^2+^ mobilization

**Fig 3:**
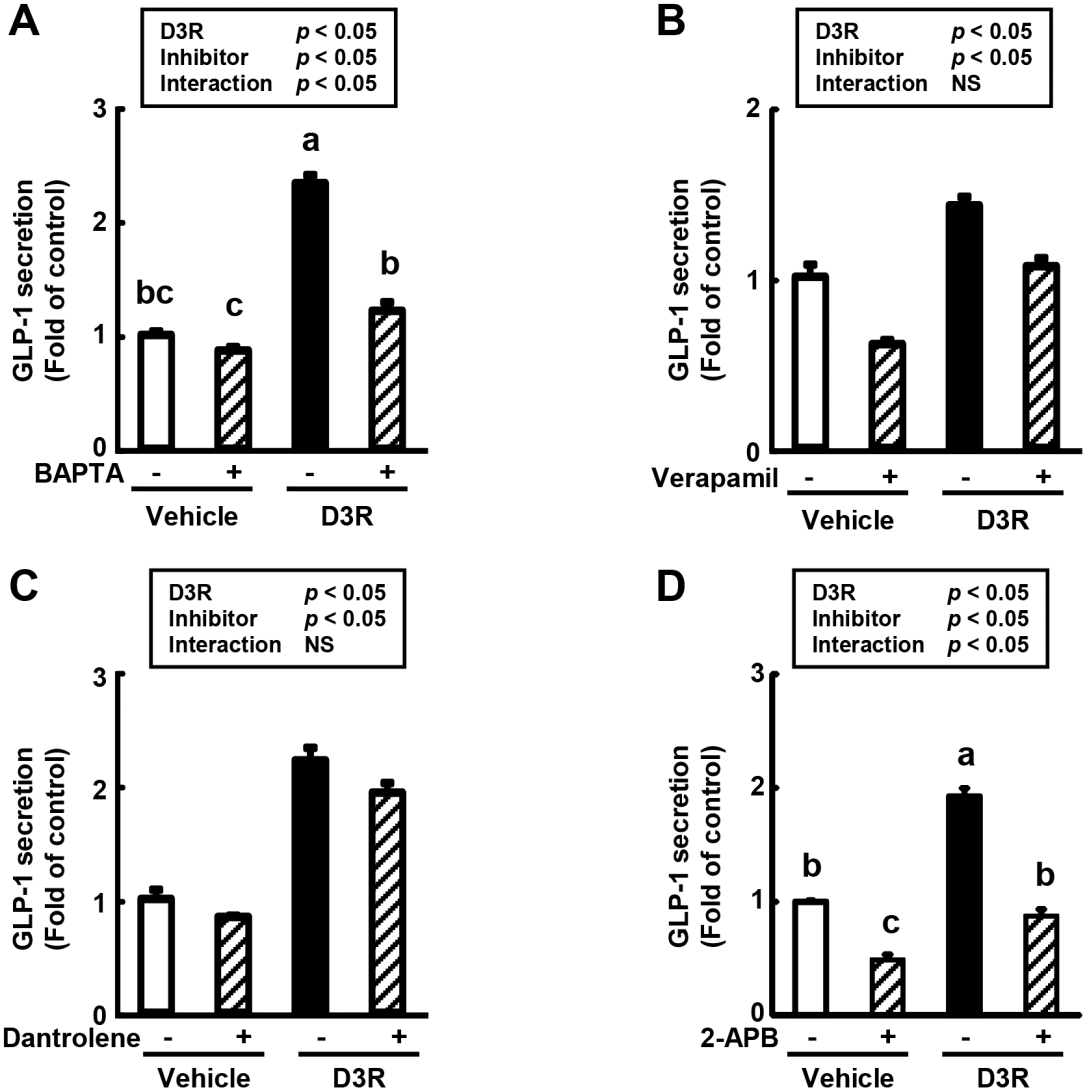
Effect of Ca^2+^ signaling pathway inhibitor on D3R-stimulated GLP-1 secretion in GLUTag cells. GLUTag cells were pre-treated with vehicle (0.1% DMSO) or (A) endogenous Ca^**2+**^ chelator (BAPTA-AM, 10 μM), (B) L-type Ca^**2+**^ channel blocker (verapamil, 20 μM), (C, D) endogenous Ca^**2+**^ channel blocker (dantrolene, 25 μM; 2-APB, 50 μM) for 15 min, followed by treatment with vehicle or D3R (100 μM) for 2 h without washing out. GLP-1 levels in the medium were measured by ELISA. Secreted GLP-1 levels are expressed as the fold change of the control levels (= 1.0). Values are expressed as the means ± SEM, n = 3. Values without a common letter (a, b, and c) are significantly different at *P* < 0.05 (Tukey-Kramer test followed by two-way ANOVA).

GLP-1 secretion occurs through exocytosis and is regulated by various signaling pathways [30–33]. In particular, increases in cytosolic Ca^2+^ levels via the opening of Ca^2+^ channels or mobilization of intracellular Ca^2+^ stores leads to GLP-1 secretion [34]. To investigate whether D3R affected intracellular Ca^2+^ levels, GLUTag cells were first treated with various Ca^2+^ fluorescent indicators (Fura2-AM: excitation at 340/380 nm and emission at 510 nm, Fluo3-AM: excitation at 508 nm and emission at 527 nm, Fluo4-AM: excitation at 495 nm and emission at 518 nm, and CaSiR-1 AM: excitation at 650 nm and emission at 664 nm), and the intracellular fluorescence was monitored during exposure to D3R. However, the modulation of intracellular Ca^2+^ levels in response to D3R administration could not be detected, because the inherent fluorescence of D3R interfered with the detection of the fluorescent Ca^2+^ indicators (data not shown). Therefore, to indirectly elucidate the intracellular Ca^2+^ response of GLUTag cells to D3R, we used BAPTA-AM (intracellular Ca^2+^ chelator) and examined the effect of intracellular Ca^2+^ chelation on D3R-stimulated GLP-1 secretion. Pre-treatment of GLUTag cells with a BAPTA-AM completely blocked D3R-induced GLP-1 secretion (Fig 3A).

To examine the GLP-1 secretion-related Ca^2+^ response triggered by D3R in more detail, the effect of various Ca^2+^ signaling inhibitors on D3R-stimulated GLP-1 secretion was investigated. In response to increasing intracellular glucose concentration, the ATP/ADP ratio increases and K_ATP_ channels close. This change triggers the opening of Ca^2+^ channels and stimulates GLP-1 exocytosis. Therefore, we examined the effect of the L-type Ca^2+^ channel blocker verapamil on D3R-stimulated GLP-1 secretion. Pre-treatment of GLUTag cells with verapamil did not affect GLP-1 secretion induced by D3R (Fig 3B).

The release of Ca^2+^ from intracellular Ca^2+^ stores is enhanced following activation of the ryanodine receptor (RyR) and IP_3_R. Here, GLUTag cells pre-treated with the RyR antagonist dantrolene did not canceled increased GLP-1 secretion after exposure to D3R (Fig 3C). In contrast, the pre-treatment of cells with the IP_3_ receptor antagonist 2-APB to inhibit intracellular Ca^2+^ mobilization via the IP_3_ receptor significantly reduced D3R-stimulated GLP-1 secretion (Fig 3D).

### Activation of CaMKII is involved in D3R-induced GLP-1 secretion

**Fig 4:**
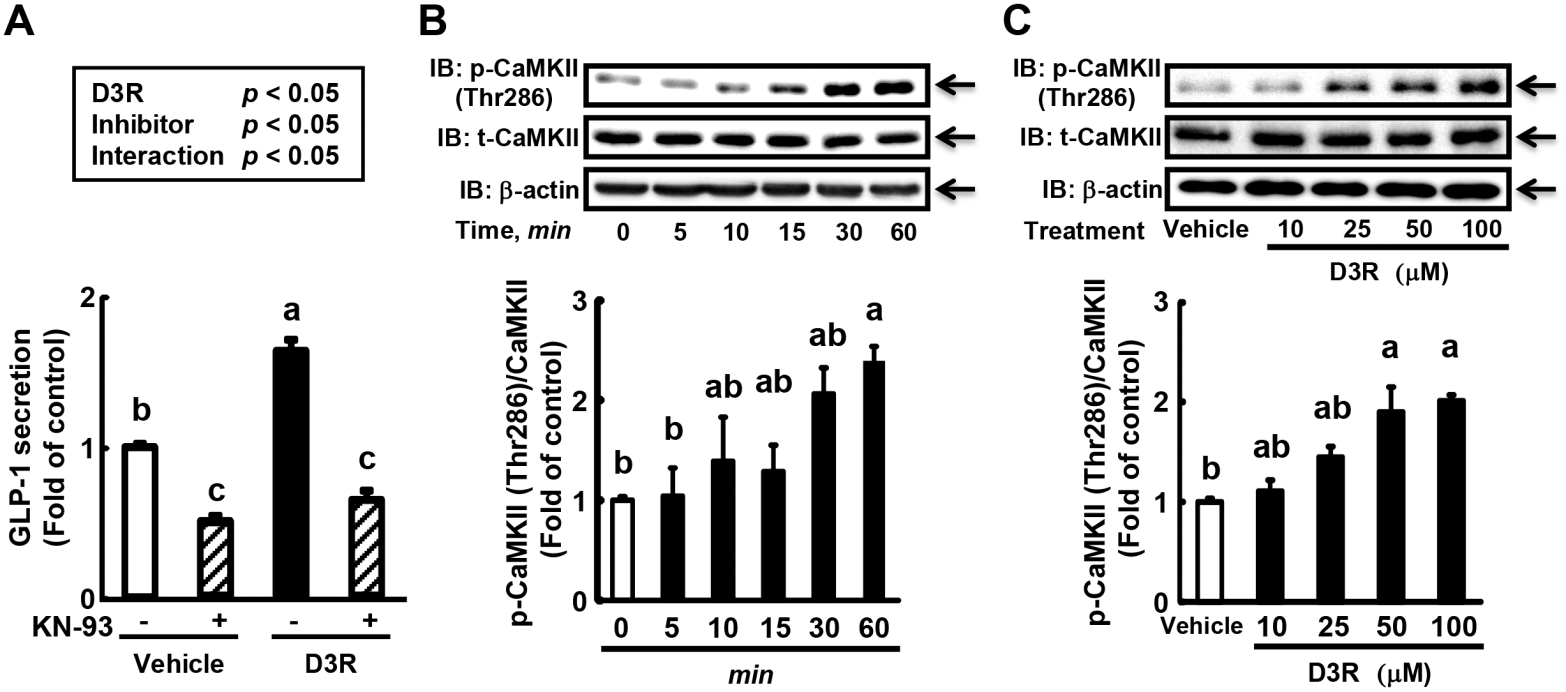
Effect of D3R on CaMKII in GLUTag cells. (A) GLUTag cells were pre-treated with vehicle (0.1% DMSO) or CaMKII inhibitor (KN-93, 10 μM) for 15 min, followed by treatment with vehicle or D3R (100 μM) for 2 h without washing out. GLP-1 levels in the medium were measured by ELISA. Secreted GLP-1 levels are expressed as the fold change of the control levels (= 1.0). Values are expressed as the means ± SEM, n = 3. Values without a common letter (a, b, and c) are significantly different at *P* < 0.05 (Tukey-Kramer test followed by two-way ANOVA). (B, C) Immunoblot analysis of the effect of D3R treatment duration (B) and dose (C) on phosphorylated CaMKII, total CaMKII, and β-actin protein. Cells were treated with 100 μM D3R for the indicated durations (B) or with concentrations of D3R ranging from 10 to 100 μM for 60 min (C). Protein intensity was expressed relative to the control (= 1.0) after normalization using the protein intensity of total CaMKII. Values are expressed as the means ± SEM, n = 3. Values without a common letter are significantly different at *P* < 0.05 (Tukey-Kramer test).

Ca^2+^ is a well-known regulator of exocytosis [35]. One of the main factors involved in Ca^2+^-mediated signaling is Ca^2+^/calmodulin-dependent protein kinase II (CaMKII), which is regulated via the binding of Ca^2+^-calmodulin, resulting in autophosphorylation of stimulatory and inhibitory sites [36]. Previous studies have shown that activation of CaMKII is involved in drug candidate-stimulated secretion of insulin from β-cells [37, 38]. As we recently demonstrated that CaMKII activation is involved in GLP-1 secretion in GLUTag cells treated with curcumin [20], we speculated that D3R-stimulated GLP-1 secretion proceeds by a similar mechanism. Therefore, we examined the effect of a CaMKII inhibitor on GLP-1 secretion induced by D3R. Pre-treatment of GLUTag cells with the CaMKII inhibitor KN-93 completely blocked D3R-induced GLP-1 secretion (Fig 4A). Based on this result, the effect of D3R on the phosphorylation of CaMKII was next examined. In GLUTag cells treated with D3R, phosphorylation of CaMKII protein was significantly induced in a time-dependent manner (Fig 4B). CaMKII phosphorylation was first detected at 5 min and continued to increase, reaching peak levels by 60 min (Fig 4B). The D3R-induced phosphorylation of CaMKII protein proceeded in a dose-dependent manner (Fig 4C). The observed phosphorylation pattern is consistent with the GLP-1 secretion profile of D3R-treated cells.

### GPR40/120 is involved in D3R-stimulated GLP-1 secretion, but not other possible signaling pathways

It is known that GLP-1 secretion is stimulated via the activation of G protein-coupled receptors (GPRs) in intestinal L cells [39]. For example, GPR40, GPR119, GPR120, and TGR5 mediate the secretion of GLP-1 enteroendocrine cells [40–43]. The coupling of GPR40/120 to the Gαq subunit leads to the IP_3_-mediated release of intracellular Ca^2+^ [42, 44], whereas Gαs-coupled GPR119 and TGR5 stimulate GLP-1 secretion via the cAMP/protein kinase A (PKA)-dependent pathway [43, 45].

In addition, recent studies have shown that GLP-1 secretagogues are stimulated by the protein kinase C (PKC) or mitogen-activated or extracellular signal-regulated protein kinase (MEK)-extracellular signal-regulated kinase (ERK) pathways [17, 46–48]. Here, to identify the mechanism underlying D3R-induced GLP-1 secretion, we investigated the possible involvement of these pathways.

**Fig 5:**
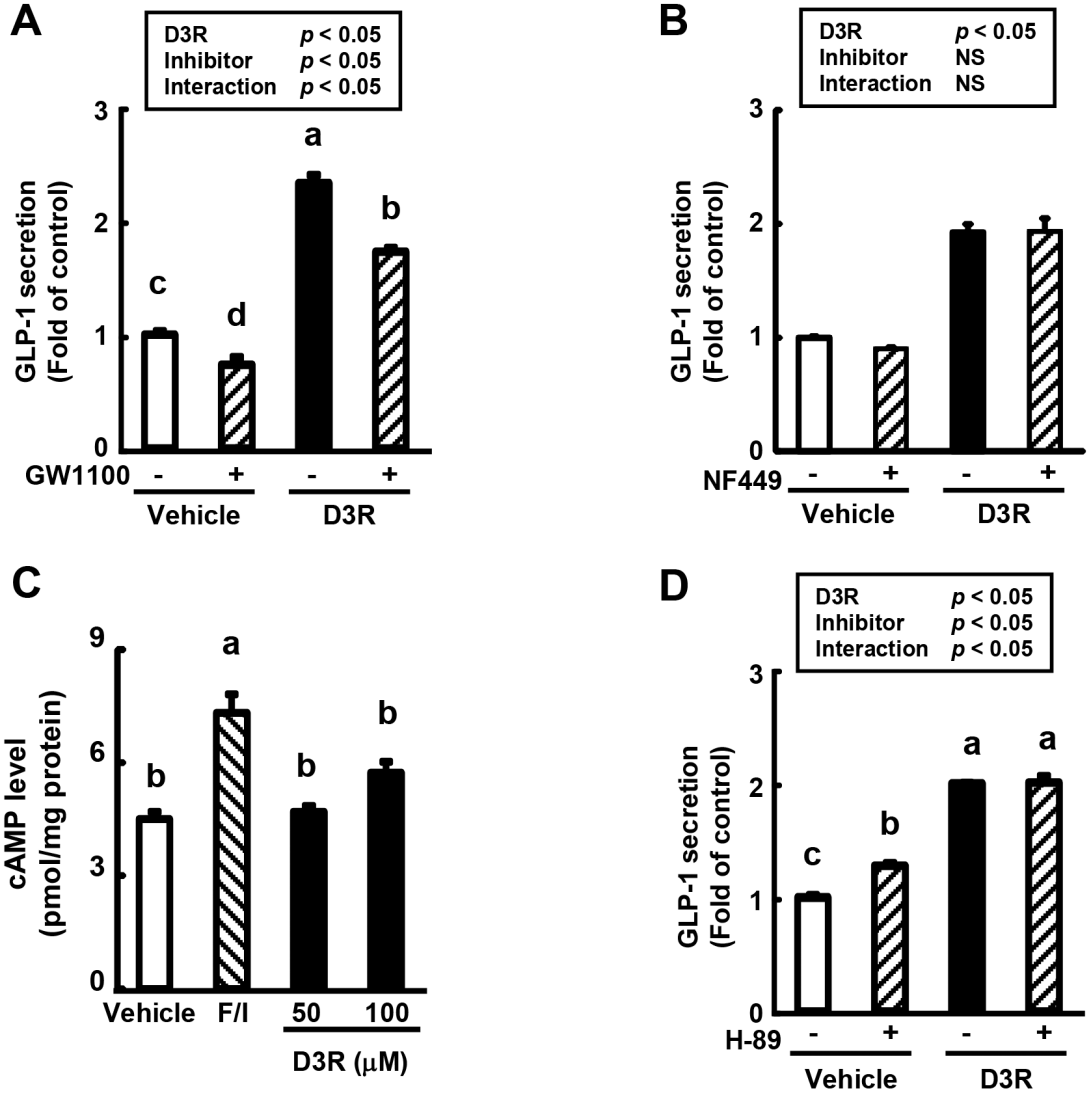
Effect of D3R on GPR signaling pathway in GLUTag cells. (A, B, D) GLUTag cells were pre-treated with vehicle (0.1% DMSO) or (A) GPR40/120 antagonist (GW1100, 10 μM), (B) Gαs subunit antagonist (NF449, 10 μM), (D) PKA inhibitor (H-89, 10 μM) for 15 min, followed by treatment with vehicle or D3R (100 μM) for 2 h without washing out. GLP-1 levels in the medium were measured by ELISA. Secreted GLP-1 levels are expressed as the fold change of the control levels (= 1.0). (C) Cytosolic cAMP concentrations in GLUTag cells treated with vehicle (0.1% DMSO), positive control (Fos, 10 μM + IBMX, 10 μM; F/I), or D3R (50 or 100 μM) after 15 min. Values are expressed as the means ± SEM, n = 3. Values without a common letter (a, b, c, and d) are significantly different at *P* < 0.05 (A, B, D, Tukey-Kramer test followed by two-way ANOVA; C, Tukey-Kramer test).

The effect of the GPR40/120 antagonist GW1100 on D3R-stimulated GLP-1 secretion was examined (Fig 5A). The pre-treatment of GLUTag cells with GW1100 significantly decreased GLP-1 secretion in D3R-treated cells compared to vehicle. In contrast, D3R-induced GLP-1 secretion was not affected by the pre-treatment of cells with NF449, a Gαs subunit-selective antagonist that serves as an indicator of GPR119 and TGR5 inhibition (Fig 5B). The treatment of GLUTag cells with D3R did not significantly affect intracellular cAMP levels (Fig 5C), and D3R-stimulated GLP-1 secretion was also not affected by the pre-treatment of cells with a PKA inhibitor (H-89; Fig 5D). In addition, pre-treatment of GLUTag cells with a PKC inhibitor (Gö6983) or a MEK inhibitor (PD98059) did not affect GLP-1 secretion induced by D3R (S1 Fig).

## Discussion

In the present study, we hypothesized that anthocyanins have multiple biological functions and aid in GLP-1 secretion, and that anthocyanins have an anti-diabetic effect through the stimulation of GLP-1 secretion. Our findings demonstrate that anthocyanins, particularly D3R, have a significant effect on GLP-1 secretion and therefore have a unique pharmacological function in the regulation of glucose homeostasis.

The treatment of GLUTag cells with various anthocyanins significantly stimulated GLP-1 secretion. The results suggested that modification of the aromatic ring with at least three hydroxyl or two methoxyl moieties is essential for stimulating GLP-1 secretion. Notably, the rutinose moiety was shown to be a potent enhancer of GLP-1 secretion, but only in conjunction with three hydroxyl moieties on the aromatic ring (D3R), as rutinose alone did not stimulate GLP-1 secretion. Interestingly, Mal3R, which contains two methoxyl groups on the aromatic ring and a rutinose moiety, did not significantly stimulate GLP-1 secretion. To our knowledge, this is the first study to demonstrate that certain anthocyanins enhance GLP-1 secretion.

In general, anthocyanins exhibit low bioavailability [49]. In this study, although 10 μM of D3R significantly stimulate GLP-1 secretion (Fig 2B), the higher levels of concentrations that were tested are not achievable in blood in vivo. However, it is not necessary for such high concentrations of D3R to be absorbed into the body and directly distributed to target cells, as these concentrations are achievable in the gut lumen, where they can exert a significant impact on enteroendocrine L cells.

To explain how D3R induces GLP-1 secretion in GLUTag cells, two molecular mechanisms are possible: 1) elevation of cytosolic Ca^2+^ via endogenous Ca^2+^ store mobilization and/or extracellular influx from L-type Ca^2+^ channels results in activation of CaMKII, which may be involved in GLP-1 secretion, as well as curcumin-induced GLP-1 secretion [20]; and 2) stimulation of the cAMP/PKA, PKCζ, or ERK pathways [17, 43, 45–48]. In the present study, we demonstrated that D3R-stimulated GLP-1 secretion is completely abolished by the pre-administration of an intracellular Ca^2+^ chelator (BAPTA-AM). Furthermore, D3R-induced GLP-1 secretion was significantly inhibited by an IP_3_ receptor antagonist (2-APB), but not by a RyR antagonist (dantrolene) or L-type Ca^2+^ channel blocker (verapamil). Taken together, these results indicate that D3R stimulates intracellular Ca^2+^ mobilization via IP_3_R.

One important target of Ca^2+^-mediated signaling molecules is CaMKII, which is regulated via binding to Ca^2+^-calmodulin, resulting in autophosphorylation of stimulatory and inhibitory sites [36]. Interestingly, evidence suggests that the activation of CaMKII in response to elevated cytosolic Ca^2+^ levels stimulates insulin secretion in β-cells [37, 38], possibly indicating that GLP-1 secretion is induced by CaMKII activation via cytosolic Ca^2+^ elevation. Supporting this speculation, we recently showed that curcumin-induced GLP-1 secretion is regulated by the Ca^2+^-CaMKII pathway [20]. As shown in Fig 4A, the pre-treatment of GLUTag cells with the CaMKII inhibitor KN-93 abolished D3R-induced GLP-1 secretion. In addition, D3R induced the phosphorylation of CaMKII in GLUTag cells in both a time- and concentration-dependent manner. Together, these results indicate that the Ca^2+^-CaMKII pathway regulates D3R-induced GLP-1 secretion in GLUTag cells. However, the exact exocytosis mechanism of GLP-1 from L cell remains unclear. A recent study indicated that vesicle-associated membrane protein 2 (VAMP2), which is involved in vesicular transport and membrane fusion, has an essential role in GLP-1 exocytosis [30]. Interestingly, binding of CaMKII to syntaxin1a, which interacts with VAMP2, is an important process in the regulation of exocytosis [50], suggesting that the D3R-mediated stimulation of CaMKII modulates VAMP2-mediated GLP-1 exocytosis.

The present study raises another question regarding the primary molecular target of D3R. GPR agonists have been shown to stimulate GLP-1 secretion in intestinal L cells [40–43]. Here, the pre-treatment of GLUTag cells with a GPR40/120 antagonist, but not a GPR119 or TGR5-related Gαs subunit antagonist, resulted in a significant decrease of D3R-stimulated GLP-1 secretion, suggesting that GPR40/120-intracellular Ca^2+^ mobilization via IP_3_R is involved in this process. Although it is also possible that D3R-stimulated GLP-1 secretion is regulated by the GPR119- or TGR5-related cAMP/PKA, PKCζ, or MEK-ERK pathways, our results do not support this conclusion.

## Conclusions

**Fig 6:**
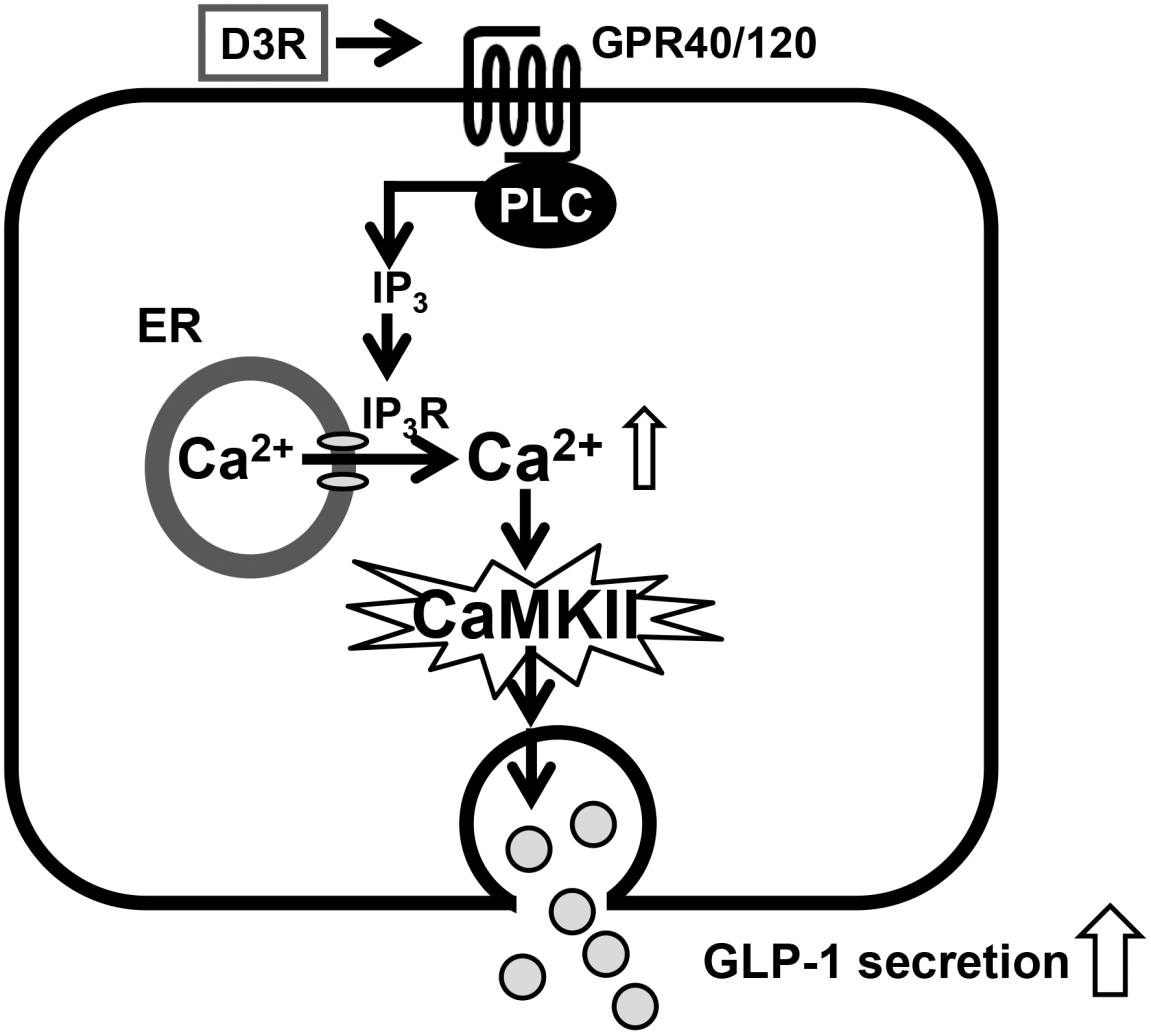
Proposed mechanism for stimulation of GLP-1 secretion by D3R in intestinal L-cells. D3R activates GPR, e.g. GPR40/120, on the L-cell surface. Activation induces IP_3_R-mediated release of intracellular Ca^**2+**^ from the endoplasmic reticulum. The elevation of cytosolic Ca^**2+**^ stimulates phosphorylation of CaMKII, and CaMKII activation leads to an increase in GLP-1 secretion from intestinal L-cells.

We demonstrated that the anthocyanin D3R significantly stimulates GLP-1 secretion in GLUTag cells through increased Ca^2+^-CaMKII pathway activation, which may be mediated by GPR40/120 (Fig 6). However, the secretion of GLP-1 is independent of the cAMP/PKA, PKC, and MEK-ERK pathways. These findings provide a possible molecular mechanism of GLP-1 secretion in intestinal L-cells mediated by foods or drugs and demonstrate a novel biological function of anthocyanins in regards to GLP-1 secretion.

## Supporting Information

S1 Fig Effect of PKC and MEK inhibitoron D3R-stimulated GLP-1 secretion in GLUTag cells. GLUTag cells were pre-treated with vehicle (0.1% DMSO) or (A) PKC inhibitor (Gö6983, 1 μM), (B) MEK inhibitor (PD98059, 50 μM) for 15 min, followed by treatment with vehicle or D3R (100 μM) for 2 h without washing out. GLP-1 levels in the medium were measured by ELISA. Secreted GLP-1 levels are expressed as the fold change of the control levels (= 1.0). Values are expressed as the means ± SEM, n = 3. Values without a common letter (a and b) are significantly different at *P* < 0.05 (Tukey-Kramer test followed by two-way ANOVA).
(TIF)
