# Supplementary figures and images for: The Anthocyanin Delphinidin 3-Rutinoside Stimulates Glucagon-Like Peptide-1 Secretion in Murine GLUTag Cell Line via the Ca2+/Calmodulin-Dependent Kinase II Pathway
Source: PLoS One. 2015 May 11;10(5):e0126157. doi: 10.1371/journal.pone.0126157 (PMC4427495; doi:10.1371/journal.pone.0126157)

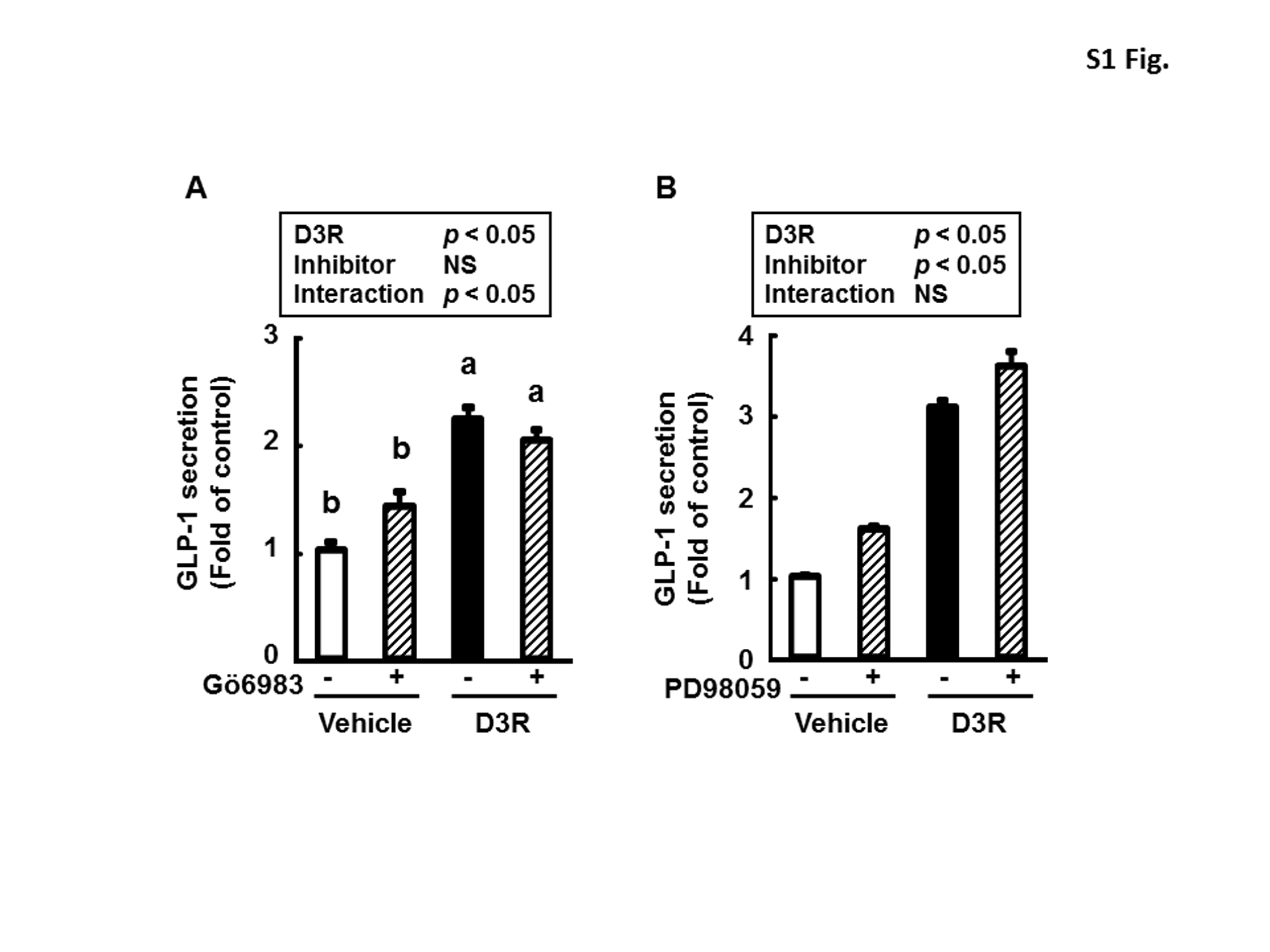

Supplement: S1 Fig — GLUTag cells were pre-treated with vehicle (0.1% DMSO) or (A) PKC inhibitor (Gö6983, 1 μM), (B) MEK inhibitor (PD98059, 50 μM) for 15 min, followed by treatment with vehicle or D3R (100 μM) for 2 h without washing out. GLP-1 levels in the medium were measured by ELISA. Secreted GLP-1 levels are expressed as the fold change of the control levels (= 1.0). Values are expressed as the means ± SEM, n = 3. Values without a common letter (a and b) are significantly different at P < 0.05 (Tukey-Kramer test followed by two-way ANOVA). (TIF) [file pone.0126157.s001.tif]
